# Limb apraxia and active inference in the visuomotor pathways

**DOI:** 10.3758/s13423-025-02852-w

**Published:** 2026-04-28

**Authors:** Riccardo Proietti, Giovanni Pezzulo, Ferdinand Binkofski, Alessia Tessari

**Affiliations:** 1 Department of Psychology, University of Bologna, Bologna, Italy; 2 Institute of Cognitive Sciences and Technologies, National Research Council, Rome, Italy; 3 Research Center Juelich GmbH, Institute for Neuroscience and Medicine (INM-4), Juelich, Germany; 4 Division for Clinical Cognitive Sciences, University Hospital RWTH Aachen, Aachen, Germany; 5 Alma Mater Research Institute for Human-Centered Artificial Intelligence, University of Bologna, Bologna, Italy

**Keywords:** Apraxia, Active inference, Dorso-dorsal stream, Ventro-dorsal stream

## Abstract

This study presents a unified neurocomputational account of apraxia within the visuomotor system using the active inference framework. In this framework, the brain encodes a generative model of the causes of sensory observations and continually reduces the surprise (or, more formally, variational free energy) associated with these observations. Our goal is to illustrate how the neuroanatomical and neurofunctional organization of the visuomotor system are emergent consequences of this minimization under the appropriate generative model, while apraxia arises from incorrect parameterizations of that model. Our active inference model of the visuomotor pathways provides a unifying perspective on key findings in the neuropsychological literature. By virtually simulating impairments in the model, we explain key aspects of five neuropsychological syndromes impacting cognitive aspects of motor behavior. This study therefore, provides a novel hypothesis about the neurocomputational basis of these pathologies and offers a formal quantitative approach to support clinical research.

## Introduction

This study aims to provide a unified perspective on the visuomotor system and its deficiencies by examining them from neuropsychological, computational, and neuroanatomical lenses. To achieve this, we employ the active inference framework. This framework suggests that the brain encodes a generative model to explain the causes of sensory observations and continuously strives to minimize the surprise (or more formally, variational free energy) associated with these observations. Within this framework, we present a computational explanation of apraxia that aligns with previous cognitive and neuroanatomical evidence.

Limb apraxia is a neurological disorder that affects the ability to intentionally perform actions, first delineated by Steinthal (1893) as impairments of planning, action execution, and movement following brain lesions. Crucially, these deficits neither stem from an inability to understand task instructions nor from issues within the sensorimotor system, object recognition, or frontal inertia (De Renzi et al., 1986; Heilman & Rothi, 1993). In most forms of apraxia, the impairment is bilateral, emerging regardless of whether the affected or unaffected hand is used (Heilman & Rothi, 2003). However, an important exception is limb-kinetic apraxia, which may present unilaterally following lesions to premotor regions (Goldenberg, 2003; Leiguarda & Marsden, 2000). The impairments can be categorized as either high level or low level, depending on the underlying cause and the specific symptoms the individual is experiencing. With respect to high- and low-level processes, apraxia is generally considered a high-level cognitive impairment as it is often associated with damage to the parietal and frontal lobes of the brain, which support high-level cognitive functions. According to Goldenberg (2014), high-level apraxia affects the cognitive and mental representation of actions, the ability to perform complex actions involving multiple steps, such as using tools or performing gestures, planning and coordinating movements. On the other hand, low-level apraxia refers to body representation and control and the details related to motion (Goldenberg, 2014). Specifically, he refers to high-level apraxia as a deficit in the cognitive or conceptual side of motor control. This type of apraxia does not reflect a primary inability to move, but rather a disruption in the abstract knowledge and planning required for an action, involving a breakdown in the ability to understand and recall the correct function or sequence of an action. This reflects a problem with the “what“ of the action. Low-level apraxia, conversely, is a deficit related to the motor or executive side of action control. Such deficit manifests as an error in the physical execution of a movement, even if the person understands the goal and concept of the action. It concerns the spatiotemporal and technical details of movement execution. This reflects a problem with the “how” of the action. Within this context, body representations play a crucial role in distinguishing between high- and low-level apraxia in terms of a dissociation between two distinct types of body representation: the body image and the body schema. High-level apraxia is related to a disruption of the body image, which is a conscious, conceptual model of one’s body (Coslett, 1998; Sirigu et al., 1991). This representation is not just about the body’s physical shape but also its spatial orientation and its relationship to the external world and objects. High-level apraxia stems from a failure to access or manipulate this abstract body representation to formulate a correct action plan, leading to problems with the “what” and “why” of action, as the conceptual knowledge of how to use their body is impaired. On the contrary, low-level apraxia is linked to a breakdown of the body schema (Gallagher, 2005; Schwoebel & Coslett, 2005), which is a preconscious, dynamic, and online sensorimotor representation of the body’s position, posture, and movement in real-time. This internal map is automatically updated through sensory feedback (proprioception, touch, etc.) and is essential for the smooth, precise execution of movements. A low-level apraxic deficit means the patient’s conceptual plan is intact, but the automatic, fine-tuned motor control necessary to carry out the movement is impaired. The body fails to accurately implement motor commands, resulting in clumsy or spatially incorrect movements.

Apraxia assessments typically involve several tasks such as imitation, tests of pantomimes, verbal instructions and pictures given the frequent coexistence of cognitive-motor and language comprehension deficits (Goldenberg, 2014; Renzi, 1985). These evaluations include both meaningful and meaningless actions, with the latter often impaired and not represented in long-term memory (Goldenberg et al., 1996; Mengotti et al., 2013; Rumiati & Tessari, 2002; Tessari et al., 2007). Meaningful actions carry recognizable meaning derived from representations stored in long-term memory and can be categorized into transitive actions, involving physical object manipulations (such as tool use or complex action sequences), and intransitive actions, encompassing symbolic gestures (such as military salutes or waving goodbye). By contrast, meaningless actions lack identifiable effects on the environment, either material or communicative, and are interpreted solely based on their kinematic aspects (Goldenberg, 2014; Proietti et al., 2023).

An early cognitive model of praxis, proposed by Rothi and colleagues (Rothi et al., 1991), was inspired by language production models (Patterson & Shewell, 1987) and was later extended by several authors (Buxbaum & Randerath, 2018; Cubelli et al., 2000; Rumiati & Tessari, 2002). This model delineates the cognitive processes involved in diverse motor tasks, including action recognition, imitation, pantomiming upon verbal command or visual cues, as well as potential processing breakdowns at different stages, depending on input-output mappings, stimulus types, and modality (Rothi et al., 1991). In the next two sections, we build on and extend this cognitive model of praxis, establishing connections with neuroanatomical substrates and computational (active inference) mechanisms, respectively.

### A functional model of visuomotor cognition and its neuroanatomical mapping

**Fig. 1:**
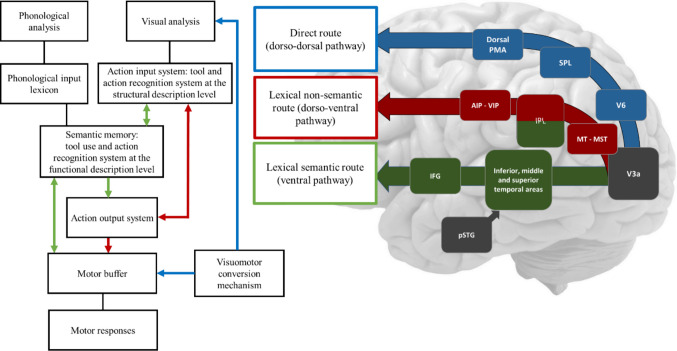
Illustration of a functional model depicting visuomotor cognition and its neuroanatomical correspondence. The top-left portion of the model represents input, comprising visual (and phonological) information. This input undergoes parallel processing through three routes, each involving varying hierarchical levels of elaboration, spanning from sensorimotor to conceptual levels. The processing stages are interconnected through three routes: a direct route (blue), a lexical non-semantic route (red), and a lexical semantic route (green). Damage at different processing stages along these routes leads to distinct behavioral deficits. On the right, these routes are mapped onto the cortical neuroanatomy, aligning with the dorso-dorsal pathway (blue for the direct route), the dorso-ventral pathway (red for the lexical non-semantic route), and the ventral pathway (for the lexical semantic route). Adapted from Tessari, Proietti et al. (2021)

Our proposed functional model of visuomotor cognition encompasses three routes that map visual inputs to motor outputs (see Fig. 1). The first route (in blue in the figure), a direct route employing a visuomotor conversion mechanism, decomposes observed actions into simpler motor components without relying on stored action representations. The direct route is neither a simple feedforward mapping from vision to action, nor a discrete module, but a dynamic process occurring within the dorso-dorsal visuomotor pathway. This process involves the brain constantly predicting the kinematic trajectory of the observed action and then adjusting its own motor plan based on the prediction error by integrating the agent’s body schema (Goldenberg, 1995, 1999, 2000; Goldenberg & Karnath, 2006; Goldenberg & Strauss, 2002). Therefore, the imitation of an observed gesture is not a simple visual-to-motor translation, but rather the process of inferring the motor commands that, given the agent’s internal body model, would generate the observed kinematic trajectory. This means that the direct route is fundamentally predictive and embodied. This route primarily supports meaningless actions (though it can alsocontribute to meaningful actions) and governs the real-time control of object-directed actions. This aspect is crucial for encoding diverse affordances, enabling rapid online processing of visual information during object interaction, particularly based on temporary features such as orientation and location (Binkofski & Buxbaum, 2013).

The second route, termed the lexical route, is specific to meaningful actions already belonging to an individual’s repertoire, and it can be further segmented into semantic and non-semantic sub-routes. The non-semantic (shown in red) aspect involves a system for recognizing actions and tools, guiding motor output (the action output system) by reproducing well-learned, meaningful actions encoded as action engrams (Tessari & Rumiati, 2002; Tessari, Mengotti et al., 2021a, Tessari, Proietti et al., 2021b). This pathway primarily relies on structure-related components of actions and tools, encompassing knowledge about stable features like typical grasping grips and action affordances (Binkofski & Buccino, 2018; Binkofski & Buxbaum, 2013; Borghi & Riggio, 2015; Cubelli et al., 2000; Pellicano et al., 2017; Rothi et al., 1991; Tessari & Rumiati, 2004). This route also fits well with the concept of technical reasoning as it proposes a distinct form of knowledge that is crucial for tool use and action: action is grounded in physical principles, not only in semantics. The ability to use tools is not solely dependent on a stored “semantic” knowledge of an object’s function (e.g., a hammer is for hammering), but relies on a more fundamental, online understanding of the physical properties of objects and their mechanical relationships. This form of knowledge is considered distinct from both the conceptual-semantic knowledge (what an object is for) and the motor-kinematic knowledge (how to move the body), and it involves mentally simulating the physical interactions between a tool and its target, a process likened to “intuitive physics.” Notably, Cubelli et al. (2000) removed the direct link between input and output lexicons in their revised dual-route model, arguing that no empirical evidence at the time supported a direct visuomotor mapping independent of semantic mediation. However, more recent neuropsychological and neuroimaging findings have provided support for the existence of a functionally distinct, non-semantic route for action imitation. Studies by Peigneux and colleagues (2004), Goldenberg and colleagues (2009), and Buxbaum et al. (2014) demonstrated that patients with conduction apraxia exhibit selective impairments in gesture imitation despite preserved semantic and conceptual knowledge, suggesting that a visuomotor transformation system can operate independently of conceptual mediation. Moreover, evidence from functional imaging (Lingnau & Downing, 2015; Rumiati et al., 2005) indicates that the parieto-frontal mirror network supports a direct perceptuo-motor mapping that enables imitation of both familiar and meaningless gestures.

The third route, the lexical semantic route (in green in the figure), encompasses more abstract components , including an action vocabulary (action semantics) and higher-level conceptual representations. This pathway facilitates the storage of motor programs through an explicit gesture identification process and semantic representations of tools, emphasizing invariant features such as function and stable affordances, and relying extensively on object knowledge (Bartolo et al., 2001; Buxbaum & Randerath, 2018; Cubelli et al., 2000; Mengotti et al., 2013; Rumiati & Tessari, 2002; Sakreida et al., 2016; Tessari et al., 2007). All pathways converge in a motor working memory buffer which temporarily retains both meaningful and meaningless motor plans for execution (Buxbaum & Randerath, 2018; Cubelli et al., 2000; Rumiati & Tessari, 2002) and facilitates learning new actions through bidirectional interactions with long-term memory (Ottoboni et al., 2021; Tessari et al., 2006).

The three routes interact closely and typicallyplay a synergistic role in visuomotor processing, depending on task demands and environmental context (e.g., Buxbaum & Kalénine, 2010; Tessari & Rumiati, 2004). However, in cases like brain damage, these routes’ distinct impacts on action reproduction become evident. For instance, patients may show specific difficulties in imitating either meaningful or meaningless actions, highlighting the relative independence between the direct and lexical routes (Bartolo et al., 2001; Cubelli et al., 2006; Mengotti et al., 2013; Tessari et al., 2007). Our simulations in the following sections illustrate how the location of impaired processing stages relates to specific cognitive and behavioral deficits (Bartolo et al., 2001; Heilman et al., 1982; Sirigu et al., 1995).

The cognitive model shown in Fig. 1 aligns with established divisions of the brain’s visuomotor pathways at a neuroanatomical level (Binkofski & Buxbaum, 2013; Rizzolatti & Matelli, 2003), building upon the foundational work of Goodale & Milner, 1992; Jeannerod et al., 1995; Mishkin et al., 1983.

The first, the dorso-dorsal stream, is specialized for online sensorimotor representations of the postural alignment of different body parts and converts physical properties of objects, such as location or size, into appropriate motor commands to control reaching and grasping. The second, the ventro-dorsal stream, contains long-term structural representations about skilled actions, known motor programs, the manipulation of known tools, and the spatio-temporal aspects of actions (Binkofski & Buxbaum, 2013; Dressing et al., 2018; Hoeren et al., 2014; Niessen et al., 2014), and also allows for technical reasoning (Bluet et al., 2025; Reynaud et al., 2019). The third, the ventral stream, processes the categorical relationships between perceptual and semantic elements (Dressing et al., 2018; Klein et al., 2013; Lambon Ralph, 2014; Musso et al., 2015; Patterson et al., 2007; Rijntjes et al., 2012; Weiller et al., 2011), including conceptual, functional, and semantic aspects of tool use and gestures, as well as more symbolic actions (Dressing et al., 2018; Heilman et al., 1982; Niessen et al., 2014; Ottoboni et al., 2021; Rijntjes et al., 1999; Tessari et al., 2007; Vry et al., 2015). This stream overlaps with areas involved in communicative tasks (Finkel et al., 2018). It represents a “gate” to the action system for the phonological input lexicon, which enables action execution in response to verbal command (Friederici et al., 2000).

Anatomically, the dorso-dorsal pathway runs from V3a to V6 to V6a and the medial intraparietal area (MIP) in the superior parietal lobule (SPL), and from there, via the superior longitudinal fasciculus II, to premotor areas (F2vr and F7-non-SEF1) (Dressing et al., 2018; Hoeren et al., 2014; Martin, Beume et al., 2016a, Martin, Dressing et al., 2016b, Martin, Nitschke et al., 2016c; Vry et al., 2015). The ventro-dorsal pathway runs, via arcuate and superior longitudinal fasciculus III, from the medial superior temporal area (MT/MST) to the inferior parietal lobule (IPL), and then to the ventral premotor cortex (AIP – F5 and VIP – F4) (Binkofski & Buxbaum, 2013; Kalénine et al., 2010; Kreher et al., 2008; Vingerhoets, 2014; Vry et al., 2012). The ventral pathway runs through the extreme capsule, the inferior fronto-occipital fasciculus and the uncinate fasciculus, connecting the inferior, middle, and superior temporal area and the IPL to anterior inferior frontal gyrus (IFG) on the left side in humans (Catani et al., 2002, 2005; Hamzei et al., 2016; Makris & Pandya, 2009; Rijntjes et al., 2012; Saur et al., 2008; Weiller et al., 2011, 2021). Moreover, it is connected with the left posterior superior temporal gyrus (pSTG), a region of the language-processing network, involved in the perception and processing of speech sounds (phonological input lexicon), mapping auditory input to phonological representations (Fiebach et al., 2002; Friederici et al., 2000). Some authors suggest that the ventral stream should also include the lateral occipitotemporal cortex (LOTC) (Lingnau & Downing, 2015; Wurm et al., 2017; Wurm & Caramazza, 2022). Wurm and Caramazza suggest that LOTC is a key region for representing actions at an abstract, object-independent level, involving a more cognitive, perceptually driven component within the LOTC with a specific role in decoding the visual features of action kinematics, independent of the objects involved. In particular, they propose that the ventral stream contains a subdivision into a ventral pathway in the ventral occipitotemporal cortex (VOTC) that specializes in recognizing object features such as colour and texture, and a lateral pathway in LOTC that is crucial for action recognition, hierarchically organized, with a posterior-to-anterior gradient that transforms perceptual information about actions into more conceptual representations. The latero-ventral stream provides a link between pure semantic processing in the infero-temporal stream and the processing of action-related components of object interactions, and is also involved in action analysis, thus providing a crucial information channel between the ventro-dorsal and the infero-temporal streams (Wurm & Caramazza, 2022). In a recent paper, Stoll et al. (2024) propose an attempt to bring in a holistic picture the different sub-streams and their role in visuomotor processing. They suggest that the latero-ventral stream plays an important role in social communication (processing biological motion), and is crucial for interactions with the environment. In particular, the “lateral pathway” (Pitcher & Ungerleider, 2021) has also been suggested to be specific for dynamic social perception and projecting into the superior temporal sulcus (STS), providing a crucial framework for understanding how the brain processes actions and intentions in a social context. This is in line with the work from Bartolo and Ham (2016) in which they propose that meaningful intransitive gestures depend on either long-term memory or social cognition, with pantomime (gestures that mimic tool use without a tool) also seen as a communicative task. The work by Rounis et al. (Rounis et al., 2024; Rounis & Binkofski, 2023) further supports this by showing altered connectivity within this network in patients with post-stroke apraxia. The finding of an anterior-to-posterior gradient within the LOTC (Metaireau et al., 2024) may reflect a sensitivity to the meaning of actions, with a posterior-to-anterior flow for temporal integration, where posterior regions process detailed perceptual information about body movements, and this information is integrated into more abstract, anterior representations of action meaning and goals.

The direct route and the processing of new movements for meaningless gesture imitation have been associated with the dorso-dorsal stream (Binkofski & Buxbaum, 2013; Hoeren et al., 2014; Martin, Dressing et al., 2016b; Rumiati et al., 2005; Tessari et al., 2007), while the processing of known gestures and the lexical routes have been related to regions of both the left ventral and ventro-dorsal streams (Binkofski & Buxbaum, 2013; Dressing et al., 2018; Kleineberg et al., 2018; Rijntjes et al., 2012; Rumiati et al., 2005; Tessari et al., 2007; Weiller et al., 2009, 2011; Wurm & Caramazza, 2022). Specifically, the ventral stream might decode the meaning of a movement at the conceptual level, with a focus on social and intransitive gestures and non-motor aspects and task-irrelevant object properties (Bracci et al., 2017; Cubelli et al., 2000; Petreska et al., 2007; Wurm et al., 2017; Wurm & Caramazza, 2022), and the ventro-dorsal stream the structural descriptions of tools and recognition of gestures as meaningful (see also Dressing et al., 2018; Tessari, Mengotti et al., 2021a, Tessari, Proietti et al., (2021).

The recent neuroanatomical models for apraxia, informed by advanced lesion-symptom mapping and connectivity studies, move away from simple, linear, stepwise pathways toward a more complex and dynamic understanding of how the brain executes actions. They suggest the brain’s action networks do not operate as rigid assembly lines, but rather rely on parallel and gradual processing, and emphasize that multiple processes such as visual recognition, semantic understanding, and motor planning occur simultaneously and influence each other continuously (Stoll et al., 2024). The model integrates the well-known dual-stream hypothesis (dividing the brain into a dorsal stream for “where/how” actions and a ventral stream for “what” recognition) but adds complexity by introducing sub-parcellations and extensive white matter connections. The model features multiple, interconnected loops that constantly exchange information. This complexity helps explain why apraxia manifests in so many different ways (e.g., struggling with imitation versustool use). Crucially, this view suggests that apraxia is not a single deficit but a continuum of symptoms that exist at the intersection of perception, motor control, and higher-level cognition. A deficit in one specific sub-pathway will result in a distinct clinical profile of apraxia. In short, the neuroanatomical model is now a highly interconnected network where the severity and type of apraxia are determined by exactly which connections within the visual, semantic, and motor-processing streams are damaged, highlighting the brain’s critical reliance on network integrity for smooth action execution (see Stoll et al., 2024, for a new integrated five-route model). This distributed, recurrent organization naturally aligns with computational frameworks such as active inference, which formalize perception and action as intertwined aspects of the same predictive process. Within this perspective, each cortical area exchanges prediction errors and beliefs about hidden causes with others in the network, and coherent behavior emerges from the joint minimization of uncertainty across these loops.

### An active inference perspective on visuomotor cognition and apraxia

Here, we characterize visuomotor cognition and its impairments in apraxia, using the active inference framework (Parr et al., 2022). Active inference starts from the premise that the brain constructs a statistical representation, known as a generative model, of what causes sensory observations. The model allows the brain to predict the world’s state and choose actions that align with expected observations, reducing surprises or deviations from predicted observations (more formally, minimizing variational free energy; see later). According to the active inference framework, agents act to minimize surprise, that is the discrepancy between expected and observed sensory inputs, by continuously updating their internal models or by acting on the world to make it more predictable (Proietti et al., 2023). In our framework, we propose a more biologically plausible account of action observation and execution, emphasizing their parallel and interactive nature. Our central claim is that the traditional, serial and hierarchical model of cognition, a serial process progressing from perception to understanding to action, is inadequate to capture the brain’s dynamic functioning. Unlike this classical feed-forward view, in active inference the brain operates as a recursive Bayesian system, constantly generating top-down predictions about sensory input and minimizing the incoming bottom-up prediction errors through two complementary processes: perceptual inference (updating internal beliefs) and active inference (acting to fulfill predictions). Within this framework, observation, understanding, and execution are not discrete sequential stages but aspects of a continuous, closed loop. Perception is inherently predictive: the brain interprets sensory information in light of its generative model, and any mismatch between predicted and actual input constitutes a prediction error that refines this model, which we may intuitively refer to as understanding. At the same time, action entails another means of prediction-error minimization: rather than executing motor commands issued by higher cognitive levels, movements emerge directly from the brain’s attempt to make its predictions become true in the external world. This bidirectional and parallel organization of perception and action offers a more realistic account of sensorimotor control and cognitive processing. In contrast to the classical serial model, the active inference framework conceptualizes the brain as a predictive system engaged in a continuous cycle of observation, inference, and action.

The specific characterization of the generative model can help us understanding impaired brain functions since any neuropsychological disorders entail a set of prior beliefs that render a patient’s behavior Bayes optimal: this means that a patient’s non-adaptive behavior is explained not by a broken inference process, but is due to damage to the biological substrates that encode priors and generative model parameters. The question we pose is which prior beliefs generate that optimal behavior and which neural structure may encode those priors (see Parr et al., 2018, for the notion of Bayes optimal pathology).

**Fig. 2:**
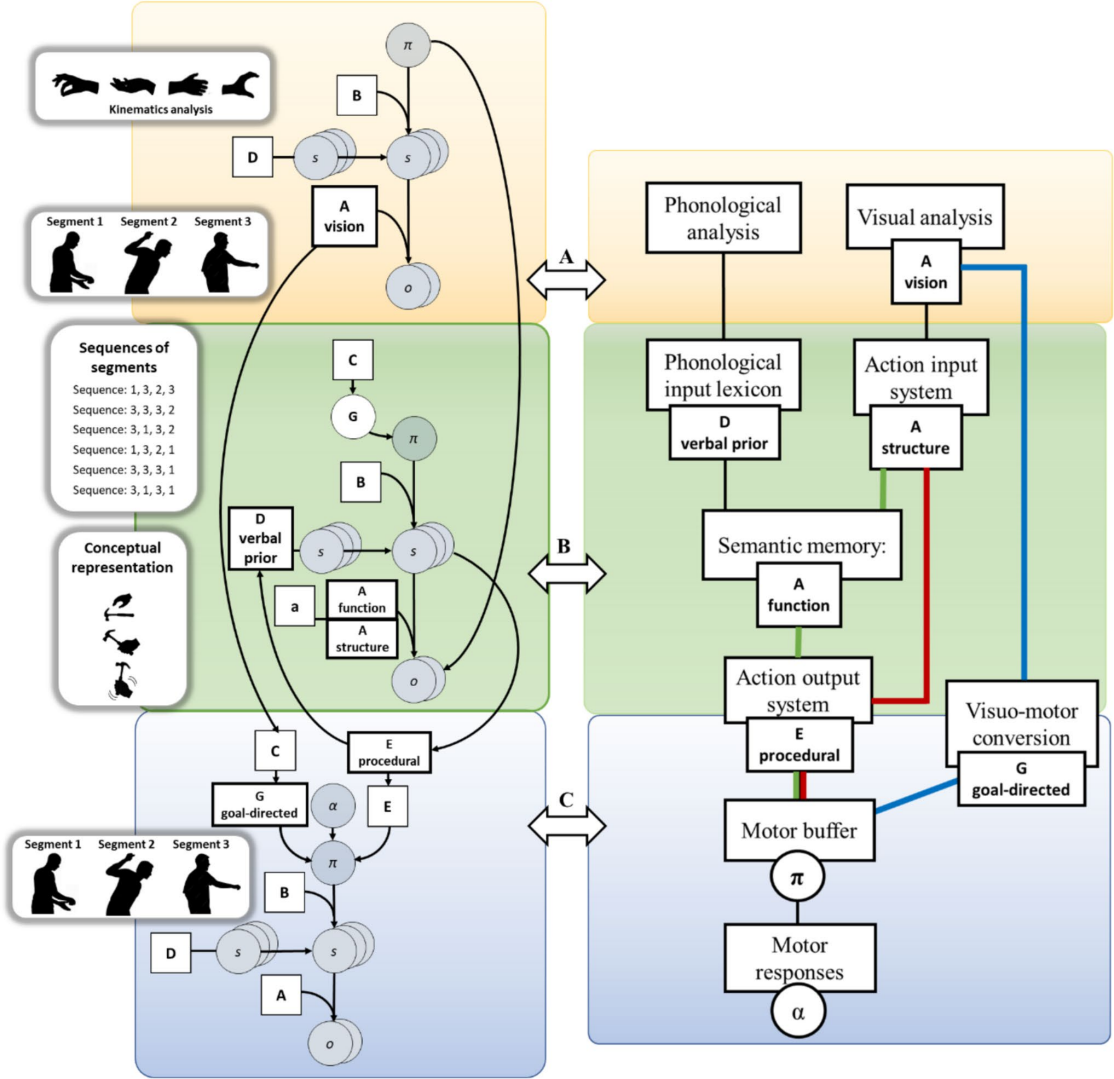
Generative model for hierarchical action processing and its mapping to visuomotor cognition. The left part of the figure shows a generative model that supports visuomotor tasks in active inference. The same model is used in Proietti et al. (2023) to simulate the understanding and imitation of actions in the context of tennis. It is formalized as a Partially Observable Markov Decision Process (POMDP), in which hidden states (s) and observations (o) are discrete probability distributions and A, B, C, D, and E matrices specify prior values and probabilistic mappings between states and observations;the α parameter specifies action precision. See the main text for an explanation of these variables and Proietti et al. (2023) for further details. The right part of the figure shows the mapping between the generative model and the functional model illustrated in Fig. 1. The generative model is hierarchical and comprises three interconnected levels, shown in the yellow, green, and blue panels. The action observation level (in yellow) maps observed actions from kinematic analysis to movement segments. The action understanding level (in green) maps from sequences of movement segments (structural level) onto conceptual representations of actions (functional level). Finally, the action execution level selects motor responses (e.g., imitative responses). Action recognition relies on the reciprocal connections between the action understanding and the action observation levels. The action understanding level generates hypotheses about the observed movements and these are tested at the action observation level; in the simulations reported in Proietti et al. (2023), this hypothesis testing operates by directing saccades to the most informative portions of movement. At the same time, posterior beliefs about movement segments at the action observation level become observations at the action understanding level, therefore creating bidirectional interactions between the two levels. This also implies that hidden state transitions at the two levels operate at different time scales: those at the action understanding level operate more slowly, as they accumulate observations provided by the action observation level. The action execution level proceeds at its own pace, independent of the other levels.

Figure 2 illustrates how the functional model of visuomotor cognition in Fig. 1 can be implemented as a hierarchical generative model in the framework of active inference. The model comprises three main levels, the action observation (yellow panels), the action understanding (green panels), the and action execution (blue panels). The three levels operate on action representations at different hierarchical levels (Grafton & Hamilton, 2007; Proietti et al., 2023; Wurm & Lingnau, 2015). The first, the action observation level, realizes a mapping from the kinematic features of the observed movement to movement segments. Considering the case of a person observing another individual hammering a nail, this level maps from observed hand configurations to representations of movement segments involving multiple body parts, such as grasping the hammer, lifting it, and striking the nail.

The secondthe action understanding level, realizes a mapping from specific action sequences to conceptual action representations. Continuing the hammering example, this is the mapping between sequences of segments recognized at the lower level that achieve a motor goal (e.g., hammering a specific nail by performing a specific sequence of segments, like grasping, lifting, hitting) and the conceptual representation of the act of hammering, which can be achieved in principle by multiple sequences of segments (e.g., first lift and then hit, or hit twice before lifting again, and so on). This is also a mapping from structural to functional aspects of action. Importantly, this mapping does not operate solely on the kinematic features of body movement, but also integrates contextual and semantic information that gives meaning to the observed action. In the hammering example, understanding the act does not derive exclusively from perceiving the configuration and movement of the hand and arm, but from inferring how these movements relate to the surrounding objects and their affordances such as the hammer, the nail, and the surface being struck. These contextual elements are part of the observed sensory data and play a fundamental role in constraining inference about the hidden causes of the observed scene (Badets & Osiurak, 2017; Federico et al., 2022; Osiurak, 2014; Osiurak et al., 2011, 2017; Reynaud et al., 2016, 2019).

In our model, we adopt a hierarchical distinction between *movement segments* and *sequences of segments* that reflects different levels of abstraction in action representation. A *movement segment* is defined as a primitive, context-free kinematic element, such as reaching, grasping, or twisting, that constitutes a basic motor unit of action. These primitive elements correspond to low-level, continuous trajectories processed by the visuomotor conversion mechanisms and the ventral visuomotor pathway and are grounded in the kinematic organization of the motor system. A *sequence of segments*, by contrast, refers to a goal-directed combination of such primitive elements. It represents a higher-level, conceptual structure defined not merely by temporal concatenation but by its purpose or intended outcome (e.g., *to hammer a nail* or *to serve a tennis ball*). This abstraction transforms individual movement segments into meaningful, goal-oriented actions (Proietti et al., 2023). This operational distinction aligns with neurophysiological evidence for multiple timescales and hierarchical levels of action representation: the anterior intraparietal sulcus (aIPS) and premotor cortex are primarily engaged in representing the kinematics of individual movements, whereas the inferior parietal lobule (IPL) and lateral prefrontal cortex are involved in encoding the structure and goals of action sequences (Fagg & Arbib, 1998; Fogassi & Luppino, 2005; Goldenberg, 2014; Grafton & Hamilton, 2007; Hamilton & Grafton, 2008; Jeannerod, 1997). Within our hierarchical model, the lower (kinematic) level processes individual segments to predict the “how” of movement, while the higher (conceptual) level integrates these into meaningful sequences to infer the “why” of the action. Thus, the distinction between segment and sequence is not arbitrary or based on temporal duration, but is based on the representational level and goal dependence. -In much of the functional magnetic resonance imaging (fMRI) literature, action observation and action understanding often refer to experimental tasks: observing an action versus observing it with an explicit instruction to retrieve its goal or meaning. Here, we use these terms to denote neurocognitive processes within the predictive coding hierarchy. Action observation refers to the bottom-up integration of sensory input into structured kinematic representations, while action understanding refers to the top-down mapping of these representations onto abstract, conceptual representations of goals and intentions. These processes are reciprocally coupled: bottom-up observation provides evidence for top-down inference and top-down understanding generates predictions that refine ongoing perception. In our model, action observation and understanding are mutually dependent processes, not task conditions.

Finally, the third, the action execution level, implements motor responses, such as the imitation of the hammering action or a verbal report about it.

It is crucial to emphasize that, within the active inference framework, information does not flow unidirectionally from sensory input to motor output, but propagates bidirectionally through the generative hierarchy. This means that higher-level priors, such as conceptual and semantic representations of tool use, do not merely label or interpret observed gestures, but actively shape inference and control at lower levels. In the context of tool use, this entails understanding that the mechanical function of a tool directly constrains the prediction and selection of the appropriate movement sequences. In other words, kinematic configurations are not independent from the conceptual level; rather, they emerge as the most likely realization of higher-level expectations about the goal and structure of the action.

From a computational perspective, implementing the above visuomotor functions requires defining various model variables corresponding to the round nodes in Fig. 2. These comprise: *observations* (o), such as visual, phonological or proprioceptive sensations at the action observation level; *hidden states* (s), such as a single movement segment at the action observation level or a sequence of segments at the action understanding level, which are not directly observed but need to be inferred by integrating multiple observations; and *policies* (π), which dictate (sequences of) executed actions, such as imitative responses at the action execution level (or even saccades performed to help infer the observed actions, which are not the focus of this study).

Furthermore, implementing the above visuomotor functions requires defining matrices that define mappings between hidden states and observations, which correspond to the (A, B, C, D, and E) squares, and the α parameter in Fig. 2. The A (likelihood) matrices map hidden states and observations. Note that in this hierarchical architecture, inferred hidden states at the level below become observations at the level above. The B (transition) matrices map hidden states through consecutive time points, providing memory to the system. The C matrices are priors over observations, which play the role of a person’s prior preferences and bias goal-directed action selection by influencing the expected free energy G (see later). The D matrices correspond to prior probabilities of initial states. The E matrices are priors over the policies that define which courses of action are more likely a priori and correspond to a habitual component of action selection. Finally, the α parameter sets the *precision* on how the policy π is translated in an executed action: the higher the value the more the selected action corresponds to the planned action.

In this study, we do not specify model variables (o, s, π) or matrices (A, B, C, D, and E) of the generative model, since our main goal is illustrating how active inference conceptualizes visuomotor cognition, not implementing it. The interested readers might consult Proietti et al. (2023), who provide a specific implementation of visuomotor cognition in the context of perceiving, recognizing, and imitating tennis movements. Instead, we explain how the model variables and matrices map into the functional scheme for visuomotor cognition advanced here. These will be relevant in the subsequent sections, in which we describe how “virtual lesions” to model variables and matrices produce specific apraxia impairments.

As depicted in Fig. 2, at the action observation level (in yellow), policies π and likelihood matrices A are configured to conduct a kinematic analysis and infer movement segments (on the left), providing an account of visual analysis in the cognitive model (on the right). At the action understanding level (in green), a higher level in the generative model (on the left) involves prior beliefs about sequences of segments, which are then mapped to the phonological input lexicon node in the cognitive model (on the right). A likelihood matrix A is employed to recognize sequences of segments, mapped with the action input level (on the right), representing a structural level of description. Another likelihood matrix A is used to infer conceptual representation (on the left) and is mapped in the cognitive model with semantic memory, representing a functional level of description. At the action execution level (in blue), links are propagated from the action observation and understanding levels. Here, the prior over policies E (on the left) represents procedural action execution in the action output system (on the right), while expected free energy G (on the left) executes actions in a goal-directed way, representing a visuomotor conversion mechanism in the cognitive model (on the right).

Once all the variables and matrices of the generative model are set, visuomotor tasks such as understanding and imitating an observed hammering action can be simulated using standard active inference equations. Here, we provide a succinct introduction to the fundamental equations that active inference uses to steer perception (infer states of the world, such as which action is being performed by a demonstrator) and action (e.g., select and execute imitative responses). We refer to Da Costa et al. (2020), Parr et al. (2022) and Smith et al. (2021) for a more detailed explanation.

In active inference, both perception and action are realized by the single imperative of minimizing the so-called *variational free energy (F)* (see Eq. 1). The *variational free energy* is defined as a function of two things: an auxiliary distribution called the variational distribution (q) that represents the agent’s best (but still approximate, i.e., variational) estimate of hidden states, and current observations (o). The equation comprises two terms. The former term assesses the Kullback Leibler (KL) divergence between beliefs about hidden states in the variational distribution q and those that would have been obtained were it possible to perform exact (as opposed to variational) inference (p(s|o)). The second term is negative log evidence (or surprise) of observations ˗ ln p(o)), which scores how well the model predicts them. Importantly, from a cognitive perspective, *minimizing divergence* and *maximizing evidence* map to two complementary sub-objectives of perception and action, respectively. In this perspective, perceptual inference lowers free energy by making the approximate posterior *Q* as close as possible to the true posterior belief p(s|o). Action (despite not appearing explicitly in the equation) lowers free energy by changing sensory data to change the log evidence term.

1 $$\underset{Variational FE}{\underbrace{\mathrm{F}(q,o)}} =\underset{Divergence}{\underbrace{{\mathrm{D}}_{KL} [q(s) || p(s|o)]}} - \underset{Evidence}{\underbrace{ln p(o)]}}$$

when considering planning, an active inference agent chooses a course of actions or policy (π) that minimizes long-term free energy, termed *expected free energy* (G). The expected free energy is specific to each policy π and has two components. The first is risk, assessing the divergence between the observations expected from a policy (q(o|π)) and the preferred observations in the agent’s model (p(o)). The second is ambiguity, measuring the expected uncertainty of the model’s likelihood function (p(o|s)). Together, these components enable the agent to plan effectively, balancing between exploiting known preferences and exploring for new information. Minimizing risk guides the agent towards exploiting preferred observations, determining goal-directed behavior by aligning with its preferences. Conversely, minimizing ambiguity drives exploratory behavior, seeking information to reduce uncertainty about the world’s states.

$$\underset{Expected\ FE}{\underbrace{\mathrm{G}(\pi)}} =\underset{Risk}{\underbrace{{\mathrm{D}}_{KL} [q(o\vert\pi) \parallel p(o)]}} + \underset{Ambiguity}{\underbrace{{\mathrm{E}}_{q\left(s\vert\pi \right)}[\mathrm{H}[p(o\vert s)]]}}$$

After calculating the expected free energy (G) for each policy, it is combined with variational free energy (F) and a prior belief over policies (E). This combination is transformed into a prior distribution over policies, p(π), utilizing a softmax function.

$${\pi} =\sigma (\mathrm{lnE}-\mathrm{F}-\mathrm{G})$$

In sum, using this method, action selection is a function of: variational free energy (F), which takes into consideration the current situation but not the future; goal-directed components of action driven by expected free energy (G), which take into consideration future states and observations; and habitual components of actions (E), which take into consideration whether in the past a policy was selected in the same context or not. Table 1 shows a glossary of technical terms.

**Table 1:** Glossary of the main computational quantities used in the active inference model and their corresponding cognitive or motor interpretations. The generative model is structured around a set of matrices (A, B, C, D, and E) that encode the agent’s beliefs about hidden states, outcomes, and actions. To illustrate their function, two concrete examples are provided below table

Term/matrix	Computational role	Cognitive/motor role	Lesion corresponds to
F Variational free energy	A functional quantifying the difference between predicted and observed outcomes (a measure of model–data mismatch). Minimizing F updates beliefs and actions to improve model accuracy	Prediction error minimization: core mechanism driving perception and action; aligns internal beliefs with sensory evidence	Global deficits in adaptive behavior or perceptual inference
G Expected free energy	Quantifies the *expected* surprise under each possible policy (course of action). Balances epistemic (information-seeking) and pragmatic (goal-seeking) value. Directs goal directed control	Action selection/planning: guides decisions toward actions that both reduce uncertainty and achieve preferred outcomes.	Impaired planning, or poor goal-directed control
Precision	The inverse of uncertainty (or expected variance) in sensory or higher-level signals. Modulates the influence of prediction errors	Confidence/gain control: encodes attention or confidence in beliefs and sensory input	Deficits in attention or confidence regulation (e.g., sensory mis-weighting)
Belief	Posterior probability distribution over hidden states, updated via Bayesian inference	Perceptual inference: represents what the system currently “believes” about the causes of its sensations	Impaired or unstable perception and belief updating
π Policy	A sequence of actions or control states considered as candidates for selection	Motor plan/strategy: represents alternative action sequences available to the agent	Deficits in motor planning or decision-making
A Likelihood mapping	Encodes the probability of observing outcomes (o) given hidden states (s). In hierarchical models, outcomes at one level correspond to inferred states at the level below.	Representational/interpretive mapping: defines how patterns at one level (e.g., sensory features or lower-level beliefs) are interpreted as causes at a higher level (e.g., percepts, concepts, intentions).	Perceptual and knowledge deficits: difficulty recognizing actions or interpreting observed events.
B Transition mapping	Probability of transitioning between hidden states across time steps (sₜ₋₁ → sₜ)	Memory and sequential structure: provides temporal continuity, modelling how states evolve (e.g., learned rules for action sequences)	Deficits in ordered actions and intuitive physics
C Prior preferences	Encodes prior expectations over outcomes — preferences that guide behavior toward desirable states	Motivational/affective value: represents preferences (pleasure, reward, avoidance of pain)	Affective or motivational deficits
D Initial state prior	Encodes prior beliefs about the initial hidden state (s₀)	Contextual initialization: defines the expected starting context of a task (e.g., “I am standing at the workbench”)	Contextual disorientation: difficulty initiating actions in the appropriate setting
E Policy prior	Encodes prior probability of selecting a given policy before evaluating outcomes	Procedural/habitual memory: represents well-learned, automatic motor routines	Impaired skilled or habitual execution

1. Transition matrix (B) and memory for gestures:

*“The B (transition) matrices map hidden states through consecutive time points, providing memory to the system.”*

*For gestures, the B matrix defines the ordered segments of a skilled action. For instance, if the current hidden state is [Hand Grasping Hammer] (s*_*t-1*_*), the B matrix specifies that the next state is [Hand Lifting Hammer] (s*_*t*_*) with high probability, ensuring the correct temporal order of movement segments that constitute the motor program “hammering.”*

2 Likelihood mapping (A) and conceptual representation:

*“This impairment can be simulated by rendering the likelihood mapping highly entropic (or ‘flat’), so that the observation of a certain sequence of movement segments does not translate into a conceptual representation.”*

*For example, when observing the sequence [Grasp → Lift → Strike], a well-defined A matrix maps it to the conceptual label [Action: Hammering]. When A becomes flat, the same observation may map equally to [Action: Hammering], [Action: Scratching], or [Unknown Action], leading to a failure to form a coherent conceptual representation despite intact visual input.*

### Virtual lesions of the generative models and apraxia

Here, we discuss how performing five types of “virtual lesions” of the generative model of Fig. 2 permits reproducing distinct aspects of apraxia. In our discussion, we assume that the active inference agent plays the role of a “patient” who has to perform four types of tasks in response to requests from a “clinician,” which are used clinically to assess correct or impaired visuomotor processing (Cubelli et al., 2000).

#### Recognition of familiar actions and pantomime agnosia

**Fig. 3:**
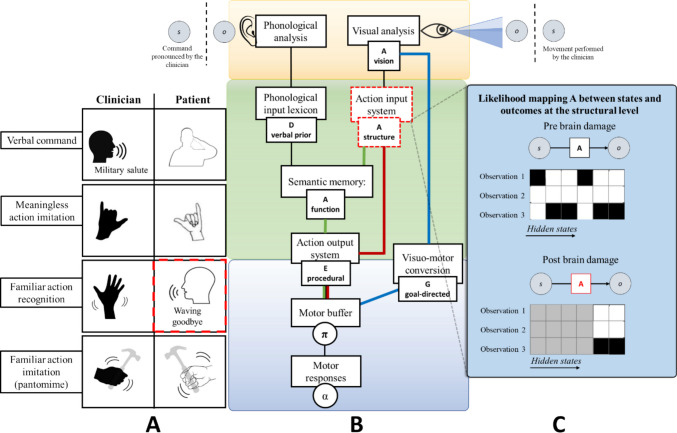
Simulation of recognition of familiar actions and pantomime agnosia. In **Panel A** shows the task of recognizing familiar actions. Red dotted lines indicate the associated behavioral impairments. In **Panel B**, the red dotted lines around the node “Action input system” indicate the locus of the virtual lesion. Here, the structural level of description is impaired. **Panel C** shows the difference between a correct likelihood function and the incorrect one, used to simulate the lesion. In the matrices, darker tints indicate stronger probabilistic relationships

The first task consists of the recognition of familiar actions. Here, the patient has to recognize the meaning of a known symbolic gesture performed by the clinician, such as a “goodbye” movement of the arm (see Fig. 3A). In our model, familiar actions are recognized through a series of mappings from the observed kinematics to movement segments (at the action observation level), then to sequences of segments and finally conceptual representations (at the action understanding level). These mappings are specified by a series of likelihood matrices (A). All these likelihood functions are correctly set in normal conditions and allow accurate action recognition across all levels. However, it is possible to create virtual lesions by impairing one or more of these likelihood matrices. Here, we illustrate the effects of impairing the likelihood function that encodes the mapping between movement segments and their sequences, representing a structural level of action understanding; see the red dotted squares in Fig. 3B. This impairment can be done by rendering the likelihood mapping highly entropic (or “flat”), so that the observation of a certain sequence of movement segments does not translate into a conceptual representation of the observed action; see Fig. 3C for a graphical illustration of the difference between a correct likelihood function (with low entropy) and an incorrect one (with high entropy). In this case, the patient has access to a correct kinematic analysis of movement and movement segments, but loses access to structural knowledge or the “meaning” of familiar actions encoded in sequences of segments, thus the patient cannot infer or verbally identify the observed action(Buxbaum et al., 2000).

The simulated patient manifests the clinical profile of *gesture and pantomime agnosia*. The first clinical report of pantomime agnosia was provided by Rothi and colleagues (1986). These authors described the case of a patient with brain lesions who was able to perform gestures when given a verbal command but could not recognize or understand the meaning of gestures pantomimed by other people. Interestingly, patients with gesture and pantomime agnosia could imitate pantomimes they could not recognise (Rothi et al., 1986). Thus, pantomime agnosia is considered a problem of access to semantics, not a damage to the semantic knowledge itself. Semantic knowledge is intact. Patients with pantomime agnosia know what objects are and their function. For example, they can verbally describe how to use a hammer and recognize the object if they see or touch it. Moreover, motor execution is intact as they can imitate a mimed gesture even if they do not understand what it means. The deficit lies in the “connection”: more specifically, the core deficit underlying pantomime agnosia does not reside within the semantic system itself, but rather in the visuomotor interface that links perceptual and conceptual levels of action representation. In our active inference formulation, this corresponds to a lesion in the action input system, specifically in the likelihood matrix (A) encoding structural knowledge about tools and gestures. Damage to this node disrupts the integration between visuomotor and semantic information, leading to a disconnection syndrome. While the semantic system, which stores knowledge about object function and use (e.g., “a hammer is for pounding”), remains intact, the patient can no longer access or apply this information to guide action or recognize gestures. The semantic content is therefore “spared” but rendered inaccessible to the motor system. This disconnection prevents the bidirectional exchange of information between kinematic and conceptual levels, corresponding to a virtual lesion in the dorso-ventral stream pathways that connect sensorimotor and higher-level conceptual representations. Consequently, the patient may still verbally describe an object’s use yet fail to pantomime its function or to recognize correct from incorrect gestures.

Our model thus provides a computational implementation of pantomime agnosia as a selective breakdown in the visuomotor-to-semantic pathway, aligning with dual-route theories of apraxia while preserving the integrity of the semantic system. This is reflected in our model since the deficit is at the level of action understanding (and more specifically, the likelihood function encoding structural knowledge) and not action observations. This observation supports the idea that the stored knowledge about gestures and tool structure and function supports their recognition and understanding but is not strictly necessary for gesture imitation and tool use. Anatomically these functions are supported by the inferior parietal lobule and adjacent regions in the temporal-parietal junction (Buxbaum et al., 1997; Lauro-Grotto et al., 1997; Negri, Rumiati et al., 2007, Negri, Lunardelli et al., 2007). Similarly, identifying an object and recognizing object-associated pantomimes can be dissociated from the ability to use tools and imitate their pantomime (Negri et al., 2007a, b; Rumiati et al., 2009; Tessari et al., 2007). In our model, performing an action that was not previously recognized is possible using the route that permits executing unknown actions: the visuomotor conversion system, illustrated in blue in Fig. 3B. In other words, in this case, the patient treats the observed gesture as an unknown, meaningless gesture. Finally, in our model it is also possible to execute gestures on verbal command (Rothi et al., 1986), because the phonological input lexicon can directly access semantic memory (see Fig. 3B). Therefore, the semantic system is “spared” in the sense that the information is still stored, but it is “inaccessible” or “unusable” for the specific task of pantomime. This explains why the patient can still access gesture knowledge in other (e.g., verbal) modalities.

#### Execution of actions upon verbal command and conceptual apraxia

**Fig. 4:**
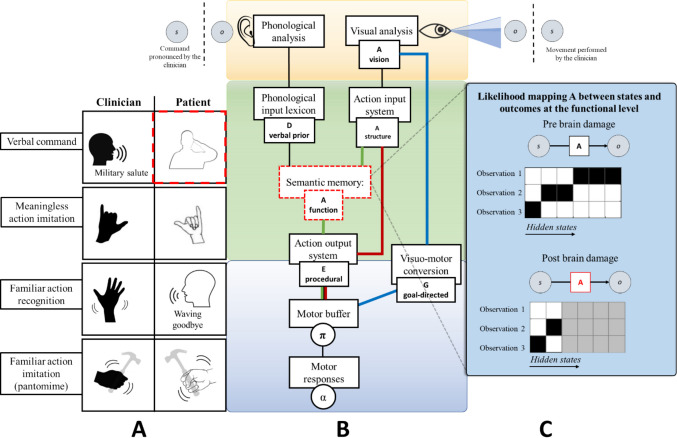
Simulation of action execution on verbal command and conceptual apraxia. In **Panel A**, the task involves the execution of actions on verbal command, where the red dotted lines indicate the associated behavioral impairment. In **Panel B**, the red dotted lines around the node “Semantic Memory” indicate the locus of the virtual lesion. Here, the functional level of description is impaired. **Panel C** shows the difference between a correct likelihood function and an incorrect one, used to simulate the lesion. In the matrices, darker tints indicate stronger probabilistic relations

The second task involves the execution of actions upon verbal command. Here, the patient has to perform an action commanded by the clinician, such as a military salute (see Fig. 4A). In our model, prompting an action via verbal command is achieved by modifying the prior belief for that action in the D vector of the sequences of segments. The recognition of this command is facilitated by mapping the conceptual representations of action at the action understanding level. This mapping is specified by a likelihood matrix (A). Under normal conditions, after the recognition of the command, the information is passed to the action execution level, where the E vector (which encodes priors over policies) is informed and prompted to execute the action in a procedural way (see Fig. 4B). However, in this context, we create a virtual lesion by impairing the likelihood mapping that allows the recognition of conceptual representations of actions. The locus of this lesion is the highest level of action and tools representation, the semantic memory, where semantic, conceptual, and functional aspects are encompassed. As before, this impairment can be achieved by rendering the likelihood mapping flat. See Fig. 4C for a graphical illustration of the difference between a correct likelihood function (with low entropy) and an incorrect one (with high entropy). This damage impairs the possibility of executing actions and pantomimes of object use based on verbal command (the phonological input lexicon is indeed directly connected to semantics).

In the simulated patient, imitation is spared (she can imitate using the visuomotor conversion mechanism, in the blue route), and there are problems in attributing meaning to gestures (i.e., recognizing) via explicit verbal identification. However, visual discrimination of existing (meaningful) actions is intact, as the action input system is spared (Ochipa et al., 1992). This occurs because the system can still provide a structural description of the input supported by the non-conceptual ventro-dorsal pathway, but the multimodal and non-motor aspects of action knowledge are impaired, supported in the posterior IPL and the ventral occipital temporal cortex (VOTC) (Bi et al., 2016; Leshinskaya & Caramazza, 2014). This recapitulates the ability to disentangle familiar from unfamiliar gestures and to tell apart well-executed from clumsy gestures but not to engage in higher-level identifications. This corresponds to the clinical profile of conceptual apraxia, also defined as a semantic type of ideational apraxia (Cubelli et al., 2000), where familiar gestures are recognized as such (the structural description is spared), but explicit identification is impaired. These deficits occur in the absence of ideomotor apraxia and semantic language impairments (Bayles et al., 1987): while ideomotor apraxic patients typically make production errors, such as temporal or spatial errors (Rothi et al., 1985), semantic ideational apraxic patients typically make content errors (Ochipa et al., 1989). The loss of knowledge concerns the appropriate actions associated with the tool: the patient shows impairment in selecting the proper tool for a particular task and understanding and solving mechanical problems based on intuitive physics (Ochipa et al., 1992). This supports the idea that the impairment locus is at a conceptual, functional description level and the neural separability of perceptual and conceptual representations (Leshinskaya & Caramazza, 2016).

**Fig. 5:**
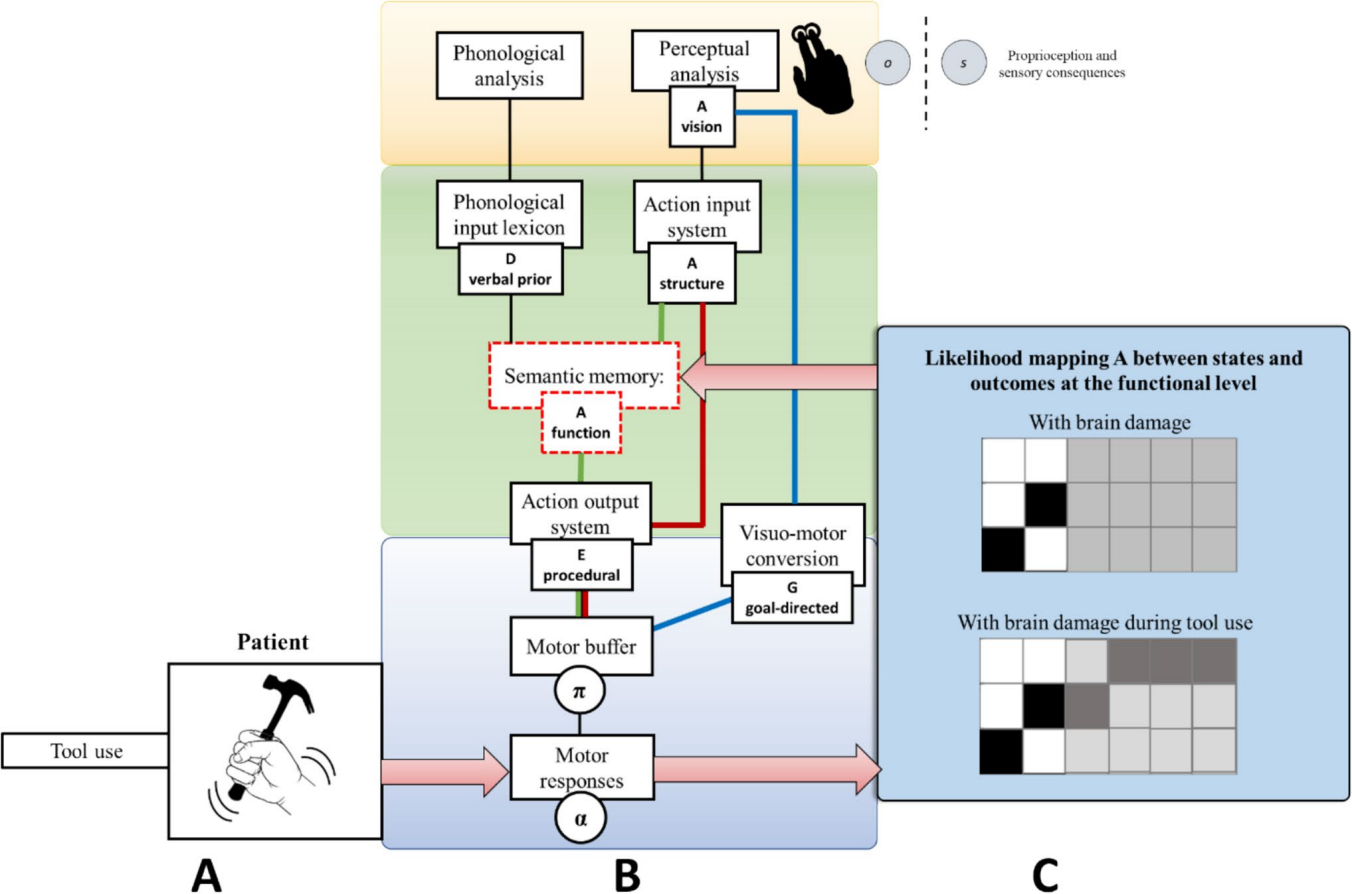
Damage to the semantic memory can be partially compensated when the actual tool use is engaged.** Panel A** shows that interactions with tools influence the node “Semantic memory” in **Panel B** by providing, as shown in **Panel C**, several sensory observations which act as evidence for the damaged likelihood matrix A

The patient can also execute an action on the visual or the proprioceptive/tactile presentation of an object, as the object recognition system (the action input system, in the red route in Fig. 4) can bypass the semantic memory and connect directly to the action output system. As anticipated, objects, their use, and the associated affordances (structural and functional; Buxbaum & Kalénine, 2010) share the encoding of their representations with movements and body parts (Maravita & Iriki, 2004). However, in the example shown, the simulated patient is not given the possibility to interact with a real object. When this occurs, the patient is exposed to a new set of sensory inputs, including proprioceptive sensations and visual and auditory consequences of using the tool. Therefore, according to our model predictions, since semantic structures consider both movements and objects, the actual use of a tool would lead to an increased temporary recovery of the behavioral impairment (see Fig. 5). This happens because the cascade of sensory input during object manipulation can positively affect impaired functional and structural knowledge. In Bayesian terms, this occurs because the posterior belief for action selection is determined by priors (which are all lesioned here) but also sensory observations that are much richer during the interaction with an object. This proves that semantic memory and the patient’s conceptual impairments are diminished (as shown in Goldenberg & Hagmann 1998; Goldenberg & Spatt, 2009; Kleineberg et al., 2018; Rothi et al., 1991). In other words, the sensorimotor aspects can partially compensate for semantic damage.

#### Imitation of a familiar action (pantomime) and procedural apraxia

**Fig. 6:**
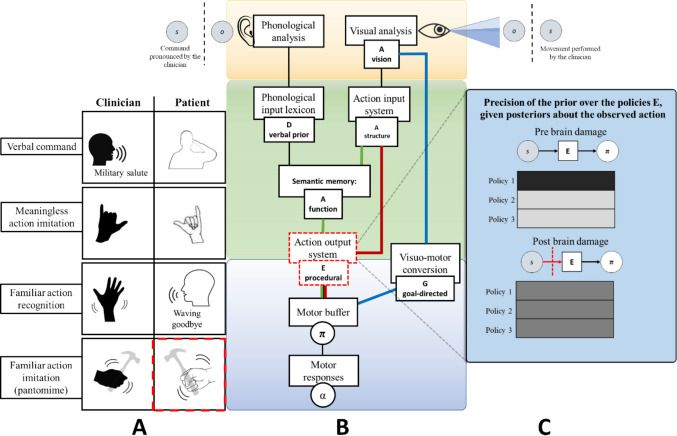
Simulation of imitation of a familiar action and procedural apraxia. In **Panel A**, the task executed is the imitation of a familiar action in which the red dotted lines indicate the associated behavioral impairment. In **Panel B**, the red dotted lines around the node “Action output system” indicate the virtual lesion’s locus. Here, the translation in a procedural action is impaired. **Panel C** shows the difference between a correct procedural policy selection and the incorrect one, used to simulate the lesion. In the matrices, darker tints indicate stronger probabilistic relations

In this task, the patient is required to imitate the execution of a familiar action (pantomime), as depicted in Fig. 6A. This imitation task relies on the connection between the information gathered during action understanding and its translation into familiar/procedural action execution – specifically, the link between posterior beliefs at the action understanding level and the skilled procedural motor controller, represented by the vector E in the action output system (see Fig. 6B).

Here, we can simulate a virtual lesion by severing this connection. By doing so, the prior over policies E is no longer informed about which policy should be selected, rendering them all equally probable (refer to Fig. 6C). In other words, the motor program stored in memory becomes disconnected from procedural-skilled motor control, which would otherwise drive the execution of the action as a cohesive action program.

The simulated patient manifests the clinical profile of a procedural type of apraxia where familiar gestures are recognized and comprehended (as semantic memory is spared), but their execution is impaired; conversely, meaningless gestures are correctly imitated through the direct route (Tessari et al., 2007). The clinical profile is supported by the observation that deficits in movement programming, which include wrong action sequences and inappropriate use of objects and matching tasks, are dissociable from more action semantics errors (Hermsdörfer et al., 2012). In our model, the term *procedural* is used in a functional and clinical sense, referring to the skilled, automatized component of action execution. While procedural memory depends primarily on subcortical structures such as the basal ganglia and cerebellum, our cortical model focuses on the cortical substrates that instantiate and use these procedural representations. The anterior IPL, the posterior middle temporal gyrus, the insula, and the extreme capsule are associated with the encoding motor knowledge as action engrams (Buxbaum et al., 2005; Heilman & Rothi, 2003; Hermsdörfer et al., 2013; Kalénine et al., 2010) to detect invariant spatiotemporal features of skilled movements shaped during motor learning (Hoeren et al., 2014; Vry et al., 2012; Weisberg et al., 2007). Within the active inference framework, these cortical representations serve as the predictive, top-down priors that implement procedural memory in action execution.

A potential paradox arises when considering imitation of familiar gestures in patients with procedural apraxia. As described, this condition is characterized by preserved comprehension of meaningful gestures but impaired execution, while meaningless gestures can still be imitated correctly. The apparent contradiction is that, if the direct (visuomotor) route is intact and can support imitation of meaningless gestures, it should in principle also enable imitation of meaningful ones, compensating for the impaired (lexical semantic and non-semantic/procedural) route. This paradox is resolved within the active inference framework by considering the automatic prioritization and competition between the two routes, as proposed by Tessari and colleagues (2004, 2007, 2021a, b). Familiar or meaningful actions activate strong, learned priors within the lexical route (semantic/procedural system), which normally dominate action selection. In procedural apraxia, these priors (E component) are degraded, producing defective motor commands that nevertheless suppress the contribution of the direct route. Because the system automatically prioritizes the lexical route for familiar gestures, the intact direct route cannot compensate, resulting in imitation failure. In contrast, meaningless gestures elicit no strong priors from the lexical route, allowing the direct route to guide imitation successfully through visuomotor transformations.

#### Imitating a novel, meaningless action and conduction apraxia

**Fig. 7:**
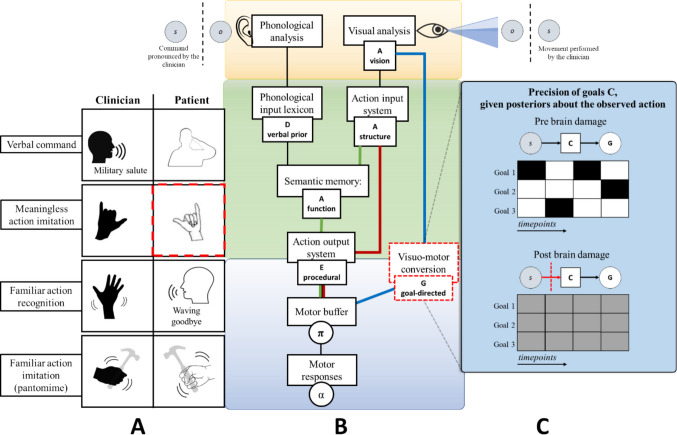
Simulation of imitation a meaningless action and conduction apraxia. In **Panel A**, the task executed is the imitation of novel, meaningless, action in which the red dotted lines indicate the associated behavioral impairment. In **Panel B** the red dotted lines around the node “Visuomotor conversion mechanism” indicate the locus of the virtual lesion. Here, the translation in a goal-directed action is impaired. **Panel C** shows the difference between a correct prior preference mapping and the incorrect one, used to simulate the lesion. In the matrices, darker tints indicate stronger probabilistic relations

In this task, the patient is tasked with imitating the execution of a novel, meaningless action, as illustrated in Fig. 7A. The success of this imitation task relies on the connection between the information gathered during action observation and its translation into goal-directed action execution – specifically, the link between posterior beliefs about movement segments and the expected free-energy goal-directed motor controller G, representing a visuomotor conversion mechanism (see Fig. 7B).

Here, we can simulate a “virtual lesion” by severing this connection. By doing so, the expected free energy for the goal-directed motor controller G will no longer attempt to move towards a preferred observation (C) since all observations will be equally preferred (a flat prior) (Fig. 7C). In other words, we disrupt the route that translates low-level, sensorimotor representations about the observed action into the motor plan (Pezzulo et al., 2018; Proietti et al., 2023).

In our anatomical model, the direct route is mapped to the dorso-dorsal stream. The simulated patient manifests the clinical profile of conduction apraxia, where the impairment is specific for meaningless actions while familiar gesture recognition, comprehension, and execution are spared (Ochipa et al., 1994). The performance of movement is often clumsy and rigid (Heugten, 1998; Shelton & Knopman, 1991). More specifically, an impaired imitation of meaningless gestures is associated with a dysfunction of the body schema (Buxbaum et al., 2000), for which encoding is based on the integration of visual and proprioceptive input about the position of body parts that support spatial limb representations encoded in the SPL (Hagura et al., 2007; Lacquaniti et al., 1995; Seelke et al., 2012) and the visuospatial transformations involved in mapping observed postures into “somatosensory spatial code” performed by the IPS (Buxbaum, 2001; Creem-Regehr et al., 2007; Watson et al., 1986). More specifically, the dorso-dorsal route is responsible for reproducing novel or meaningless actions by directly mapping observed kinematic patterns onto motor commands. This process operates independently of stored conceptual or procedural knowledge and is therefore crucial when imitating gestures that the system has never encountered before. Anatomically, this route relies on projections extending from the superior parietal lobule (SPL) to the dorsal premotor cortex, with a critical convergence point in the inferior parietal lobule (IPL), particularly in the PFG region (Area 40/Supramarginal Gyrus). The IPL serves as a key hub for visuomotor integration, transforming visual representations of movement into executable motor plans. Lesions to this region disrupt this direct visuomotor conversion, producing the characteristic deficit in meaningless imitation observed in conduction apraxia (Goldenberg, 2009, 2017; Goldenberg & Spatt, 2009; Lesourd et al., 2018). Accordingly, while the dorso-dorsal route is functionally specialized for meaningless imitation, its integrity critically depends on the IPL, which mediates the translation of visual input into motor output in the absence of semantic or goal-related context. Our generative model recapitulates this as the disruption is localized in the representations of the kinematic analysis and movement segments. Numerous clinical cases of patients able to correctly execute tool pantomime and symbolic gestures but unable to imitate meaningless actions have been reported, and some of them also reported double dissociations between imitation of meaningful and meaningless actions (Bartolo et al., 2001; Goldenberg & Hagmann, 1997; Ochipa et al., 1994; Tessari et al., 2007). These studies support the notion that imitating meaningless gestures relies on a direct visuomotor route that rapidly maps perception to action execution independently from semantic memory. Most impairments have been observed after left brain damage, specifically in the inferior parietal cortex, which is more commonly associated with apraxia (Bartolo & Ham, 2016; De Renzi et al., 1980; Goldenberg, 1995; Goldenberg & Hagmann, 1997; Heilman & Rothi, 1993; Tessari et al., 2007). Indeed, meaningless hand gesture processing predominantly comprises brain areas involved in action planning. The network includes the temporo-occipital junction (BA 19/37) and the inferior and superior parietal cortex (BA 40 and BA 5/7), with a critical role of the inferior parietal lobe for mapping perception to action (Dressing et al., 2018; Martin, Beume et al., 2016a; Martin, Dressing et al., 2016b, Martin, Nitschke et al., 2016c).

#### Simulation of all tasks and action working memory apraxia

**Fig. 8:**
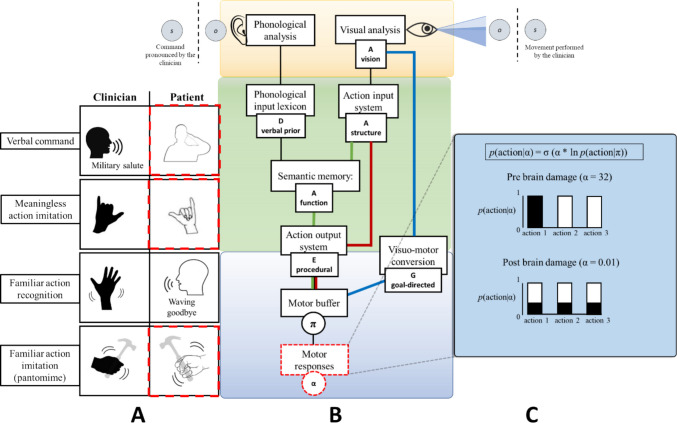
Simulation of all tasks and action working memory apraxia. In **Panel A**, all the tasks are executed. Here, the red dotted lines indicate the behaviorally impaired tasks. In **Panel B**, the red dotted lines around the node motor responses indicate the locus of the virtual lesion. Here, the translation of planned actions into executed movements is impaired. **Panel C** shows the difference between a high value of α and a low one, used to simulate the lesion affecting the action working memory buffer

In this scenario, the patient performs all the previously mentioned tasks, and we induce a virtual lesion by assigning a low value to the precision parameter (α) (Fig. 8). This parameter acts as a system translating the policy (action planning) into a sequence of actions (action execution), resulting in high randomness in selecting the latter. This parameter, analogous to a memory buffer for actions, holds the motor plan ready for implementation (Rumiati & Tessari, 2002). The higher its value, the more precisely the planned actions are implemented. Conversely, lower values can be viewed as an inability to retain the current plan and translate it into actions. When this mechanism is impaired, all motor responses (related to both known and new actions and in each presentation modality) are affected, as the motor buffer plays a role in all aspects of action execution (Fig. 8C).

The simulated patient exhibits impairments in executing gestures, whether on verbal command or in imitation tasks (both meaningful and meaningless actions). The working memory subsystem dedicated to processing motor information is distinct, not associated with verbal or visuospatial working memory, and specifically designed for encoding, retaining, and recalling goal-directed actions and body configurations (Rumiati & Tessari, 2002; Smyth et al., 1988; Galvez-Pol et al., 2020; Ottoboni et al., 2023). The existence of a working memory component for actions is supported by several behavioral studies (Rumiati & Tessari, 2002; Smyth et al., 1988; Smyth & Pendleton, 1990; Wood, 2007). These studies have observed a decreased memory span in primary motor tasks executed concurrently with a secondary motor task, compared to verbal or spatial secondary tasks, indicating the specificity of motor working memory. A recent study has also reported neuropsychological evidence for a distinct motor working memory subsystem: left hemisphere stroke patients with apraxia demonstrated a more pronounced deficit in motor working memory tasks (involving pictures of oriented objects, meaningless and meaningful actions) compared to left hemisphere patients without apraxia and controls (Bardakan et al., 2022). At the neuroanatomical level, the maintenance of this motor information seems to be associated with the frontoparietal praxis network. Increased functional connectivity and activation in the frontoparietal network during a greater memory load of motor information have been reported (Cai et al., 2018). Furthermore, the left middle and right inferior frontal gyri and the superior and inferior parietal lobule of the left hemisphere have been implicated in the maintenance of motor representations of biological motion (Lu et al., 2016). In the context of tool use, activations in the left inferior frontal gyrus and the left ventral premotor cortex have been observed during the maintenance of manipulable tools compared to non-manipulable ones (Mecklinger et al., 2002). In our model, the lower the action precision (α) parameter, the less precisely the planned actions are implemented, whether they are meaningful or meaningless actions, or object use and pantomimes. This aligns with the neuropsychological evidence provided by Bardakan and colleagues (2022), who demonstrated that the overall performance in motor working memory tasks predicted the severity of apraxic deficits in clinical tests of gesture imitation.

#### Summary of the simulated clinical profiles and corresponding model lesions

Here we show a comprehensive summary table that serves as a bridge, explicitly linking the model’s terminology to clinical terminology and computational parameters.

**Table:** 

Deficit as given in the article	Lesion pathway/Component (active inference model)	Classical clinical correspondence	Primary functional manifestation
Pantomime agnosia	Lexical non-semantic route. Lesion to Structural A mapping	Pantomime agnosia	Able to perform gestures but unable to understand the meaning
Conceptual apraxia	Lexical semantic route. Lesion to Functional A mapping	Semantic type of ideational apraxia	Unable to perform pantomime and actions on verbal command
Procedural apraxia	Lexical semantic and non-semantic routes. Lesion to the prior E over skilled (procedural) actions	Ideomotor apraxia (execution subtype)	Familiar gestures are recognized and comprehended but their execution is impaired
Conduction apraxia	Direct (visuomotor) route disconnection. Lesion to the direct connection between visual input and motor output (prior over G at the action execution level)	Conduction apraxia or apraxia for imitation (of meaningless gestures)	Inability to imitate new or meaningless gestures due to the disruption of online visuomotor conversion
Action working memory apraxia	Execution level. Loss of motor precision (α)	Spatio-temporal inaccuracy deficit	Imprecise, uncoordinated, or disorganized execution of movements, caused by uncertain motor output

## Discussion

In this study, we proposed a computational account of apraxia within the active inference framework. This account aims to offer a novel conceptualization of apraxia and address certain issues in its definition. For instance, Petreska and colleagues (2007) suggested a lack of systematicity in apraxia assessments, leading to contradictory and inconsistent results, ambiguous terminology use, and a high dimensionality in the parameters of the models, complicating statistical approaches.

In active inference, understanding brain function and dysfunction revolves around defining an appropriate generative model for the task (Parr et al., 2020; Parr & Friston, 2018). Here, we adopt a generative model of visuomotor cognition that aligns with a prior characterization of hierarchical action processing (Proietti et al., 2023). This model incorporates crucial neuroanatomical evidence, such as the existence of three routes for action processing, extensively discussed in both language processing (Coltheart et al., 1983; Coslett, 1991; Law et al., 2005; Wu et al., 2002) and the neuropsychological literature (Dressing et al., 2018; Martin, Beume et al., 2016a, Martin, Nitschke et al., 2016c).

The proposed generative model is hierarchical, emphasizing the significance of hierarchical processing not only for action understanding but also for various cognitive abilities (Grafton & Hamilton, 2007; Hasson et al., 2015; Kiebel et al., 2008; Lee & Mumford, 2003; Murray et al., 2014; Pacherie, 2008; Pezzulo et al., 2018; Proietti et al., 2025; Vidaurre et al., 2017). This hierarchical model consists of three levels, illustrated in Fig. 2. The action observation level maps kinematic movement features (elementary motor engrams and body parts relations) to movement segments, capturing relationships between body parts (see also Tessari & Ottoboni, 2022, for a discussion on the role of automatic body part ricognition in imitation). The action understanding level provides a semantic interpretation by encoding structural descriptions (sequences of movement segments) and functional-conceptual descriptions. Here, identification occurs as representations generalize at a conceptual level, becoming less constrained by motor aspects. The action execution level replicates the motor response structure, enabling the reproduction of observed or instructed actions.

The action observation and action understanding levels engage in reciprocal interaction during action observations. At the action understanding level, hypotheses about observed actions form at a slower time scale and are tested by engaging saccades at the action observation level, at a faster time scale. Simultaneously, posterior beliefs regarding postures at the action observation level ascend as messages, becoming observations at the action understanding level (Proietti et al., 2023). This reciprocal exchange implies that action processing across different levels can mutually influence each other. This is supported by extensive empirical literature demonstrating the integration of forward (motor) and inverse (perceptual) models in cortical motor hierarchies, which enable predictive motor control and perception–action coupling (Adams et al., 2013; Friston, Rosch, et al., 2017b; Kawato, 1999; Kiebel et al., 2008, 2009; Wolpert et al., 2011).

Crucially, our research demonstrates that “virtual lesions” of the model produce deficits associated with five distinct types of limb apraxia: *pantomime agnosia*, involving the loss of structural knowledge about actions and tools; *conceptual apraxia*, characterized by the loss of high-level functional representation knowledge; *procedural apraxia*, indicating the inability to translate perceived movements into pre-existing motor programs; *conduction apraxia*, where the failure in movement translation affects lower-level sensorimotor capabilities; and *action working memory apraxia*, resulting from a reduced memory span in motor tasks. These deficits emerge automatically by lesioning various parameters within the same generative model, impacting different stages of action processing. This underscores the broad applicability of our approach. Our neuropsychological characterization is inspired by the concept of Bayes optimal pathology (Parr et al., 2018), explaining a patient’s non-adaptive behavior in terms of damage to the biological substrates encoding model priors and parameters, rather than an incorrect inference process.

Furthermore, our model reinterprets the so-called direct route of imitation within the active inference framework. Rather than a simple feedforward mapping from visual input to motor output, the visuomotor conversion process is conceived as a dynamic predictive loop. In this view, imitation arises from the brain’s attempt to minimize the prediction error between observed and predicted kinematics, using internal body representations to constrain possible motor solutions. This approach reconciles the classical dual-route model (Buxbaum, 2001; Cubelli et al., 2000; Rothi et al., 1991) with more recent evidence challenging the existence of a purely “direct” route (Goldenberg, 1995, 1999, 2014; Goldenberg & Karnath, 2006). The ability to imitate thus depends on visuospatial and body-schema representations that shape motor predictions. The “directness” of the route refers not to the absence of cognitive mediation, but to the automatic and embodied nature of the predictive process that can operate without explicit semantic knowledge of the action being imitated.

This model also accommodates the long-standing debate between memory-based and reasoning-based accounts of tool use and pantomime. In traditional dual-route frameworks, impairments in tool use have been attributed either to the loss of stored motor engrams (the “action lexicon”) or to deficits in technical reasoning, the ability to infer how an object’s structure affords specific actions (Goldenberg & Hagmann, 1998; Lesourd et al., 2025; Osiurak & Le Gall, 2015). In our active inference formulation, these two accounts correspond to different hierarchical levels of the same predictive process. The action lexicon aligns with the higher, conceptual level of the generative model, which encodes priors about the function and goal of an action (e.g., “pounding with a hammer”). Technical reasoning or structural inference emerges from the generative process that links these conceptual priors to lower-level kinematic predictions. When an agent encounters a novel or ambiguous tool, the model actively infers the most plausible causal relationships between the tool’s shape and possible movements, thereby “reasoning” about its use through predictive simulation. Thus, the model does not treat lexical and reasoning-based processes as separate or competing mechanisms, but as complementary aspects of hierarchical inference: stored conceptual knowledge provides priors, while reasoning reflects the dynamic prediction and error minimization that links these priors to sensory and motor representations. In this sense, apraxic deficits can result either from degraded priors (lexical-level impairment) or from disrupted predictive mapping between conceptual and sensorimotor levels (reasoning-level impairment), offering a unified, mechanistic explanation for both forms of tool-use disturbance.

Another important feature of our generative model is its demonstration of degeneracy in action recognition and execution. In biological systems, degeneracy denotes the capacity to employ alternative structures for the same task (Edelman & Gally, 2001; Tononi et al., 1994, 1999). In the brain, this implies that a cognitive function can be supported by multiple neural systems (Friston & Price, 2003; Noppeney et al., 2004; Price & Friston, 2002; Sajid et al., 2020). This holds significant relevance in neuropsychology, where identical behavioral outcomes can arise from different strategies or pathways. In our model, degeneracy manifests as action execution being driven by the posterior belief of three factors: the sequence of movement segments, the structural description, and the functional-conceptual description. Due to the robust, parallel organization of the three cortical streams, the execution of actions can be conditionally preserved even if one pathway is damaged, often relying on compensatory, but less efficient, pathways or requiring specific sensory input to restore functionality. This leads to selective clinical impairments rather than global paralysis.

Furthermore, although our model was developed to explain apraxia, it can also account for optic ataxia (OA), a visuomotor disorder classically associated with lesions of the dorso-dorsal pathway. In the framework of active inference, both disorders can be understood as disruptions of the generative model underlying action, but at different hierarchical levels. Optic ataxia arises from lesions in the parieto-occipital junction and superior parietal lobule (Karnath & Perenin, 2005) which compromise the precision of online visual feedback used to guide movements. In our model, this corresponds to a reduction in the precision of visual prediction errors, such that the system no longer “trusts” the current visual input. As a result, the initial motor plan is intact, but ongoing correction during movement execution fails – producing the characteristic terminal errors of OA.

In contrast, apraxia reflects a higher-level deficit in policy selection or visuomotor mapping: the system fails to generate or select the correct motor plan in the first place. Thus, whereas OA involves a breakdown in sensory precision during execution, apraxia involves a breakdown in prior or policy precision during planning. This distinction explains why the two syndromes, despite arising from adjacent parietal regions, produce qualitatively different visuomotor deficits.

Moreover, our framework shares several features with existing models of action control. The Affordance Competition Model (Cisek & Kalaska, 2010) similarly views action selection as a competition among potential motor plans, biased by context and goals. In active inference, this process is formalized as the minimization of expected free energy, offering a computational account of how such biases emerge from prior beliefs and goal precision. Likewise, the Theory of Event Coding (Hommel, 2009) proposes a shared representational space for perception and action (“common coding”). Active inference provides a mechanistic realization of this principle: perception and action are both means of minimizing prediction error under a generative model. While TEC and ACM focus primarily on behavioral and neural dynamics, Active inference extends these ideas to a computational and pathophysiological level, explaining how specific disruptions can lead to clinical deficits such as apraxia.

One limitation of the current model concerns the interpretation of the “sensory enrichment” hypothesis in tool use. The model does not predict that enriched tactile feedback alone can restore performance, as experimental data show that tactile input by itself does not improve tool-use ability (Goldenberg et al., 2004). Rather, the model assumes that interacting with a real tool provides a structured, multisensory context that constrains the space of possible actions through affordances and motor priors. The real tool acts as a strong re-activation cue that increases the precision of semantic–motor inferences by narrowing the range of plausible motor policies. Thus, the compensatory effect is not purely sensory, but computational: the full multisensory feedback loop supports temporary access to degraded semantic–motor knowledge. Another limitation is that the current active inference model focuses primarily on the visuomotor and semantic–motor components of apraxia. It does not yet capture the full range of gesture deficits, such as double dissociations between pantomime of tool use and symbolic gestures (Bartolo & Cubelli, 2014; Bartolo & Ham, 2016; Baumard et al., 2025) Symbolic gestures often involve communicative and socially grounded priors that likely depend on more ventral and semantic pathways not explicitly modeled here. Future extensions of the model could incorporate these higher-order, socially embedded representational systems to provide a more comprehensive account of the apraxia spectrum.

### Future directions

While this study focused on conceptually describing the generative model and its disruptions in apraxia, a crucial avenue for future research involves leveraging this model to aid data analyses in clinical settings. Establishing generative models capable of replicating specific deficits enables quantitative computational phenotyping of patients (Schwartenbeck & Friston, 2016), aligning brain computations with measurable behaviors (Krakauer & Shadmehr, 2007; Mirza et al., 2016; Testolin & Zorzi, 2016). This direction forms part of our intended focus for future studies.

Another significant route for future research involves elucidating the neural foundations of the proposed model. Despite the seemingly abstract nature of active inference’s optimization processes, they can be linked to alterations in neural activity and connectivity across multiple levels (Bastos et al., 2012; Da Costa et al., 2021; Friston, Fitzgerald et al., 2017a; Friston & Kiebel, 2009; Parr et al., 2022). For instance, processes such as perceptual inference correspond to changes of synaptic activity, while learning and optimizing the generative model relate to modifications in synaptic connections and their effectiveness. Attention and precision signal optimization are associated with fine-tuning post-synaptic gains, and behaviors and motor control involve refining proprioceptive prediction errors. These mechanisms collectively work toward minimizing free energy or, equivalently, maximizing evidence for the organism’s generative model. Notably, lesions within our model align with the concept of the disconnection syndrome (Catani & Ffytche, 2005), which stems from disruptions in the likelihood mapping between two neural populations, represented in our model’s Dirichlet (a) parameters of A matrices. At the neural level, these parameters correspond to synaptic connection strengths, where incorrect Hebbian neural plasticity might contribute to such a disconnection. Moreover, a disconnection can occur when the propagation of posteriors between different hierarchical levels is disrupted, resembling interruptions in axonal connections. Further development and empirical testing of these concepts are crucial objectives for the future.

The provided computational framework could help reduce theoretical noise by providing a clear, predictive structure for complex clinical patterns. Acting as an organizing principle, it shows that seemingly disparate deficits are simply the result of damage to different nodes or connections within the same underlying three-route architecture (lexical semantic, lexical non-semantic, and direct). By associating a specific label (e.g., “procedural”) with a specific computational parameter (e. g., prior over habit E) and a specific neural pathway (e. g., lexical non-semantic), the model makes testable, quantitative predictions about which tasks will be spared and which will be impaired, thereby aiding future clinical research and differential diagnosis. In essence, we are using these specific terms as tools for scientific description within the model, rather than as proposals for new entries into the clinical nosology. The goal is to clarify the function of the underlying neurocognitive system, which ultimately supports and informs clinical labelling.

## Data Availability

Not applicable to a theoretical paper.

## Code availability

Not applicable to a theoretical paper.
